# Assessment of Macular Function Following Internal Limiting Membrane Peeling With ILM Blue®

**DOI:** 10.7759/cureus.11873

**Published:** 2020-12-03

**Authors:** Annekatrin Rickmann, Sami Al-Nawaiseh, Maria Della Volpe, Torsten Straßer, Lukas Bisorca-Gassendorf, Peter Szurman, Kai Januschowski

**Affiliations:** 1 Ophthalmology, Knappschaft Hospital Saar, Sulzbach, DEU; 2 Ophthalmology, University of Basel, Basel, CHE; 3 Ophthalmology, University Eye Hospital Tübingen, Tübingen, DEU

**Keywords:** ilm blue, ilm peeling, brilliant blue g, epiretinal membrane

## Abstract

Purpose

To evaluate clinical outcome after surgery of idiopathic epiretinal membranes (ERM) with internal limiting membrane (ILM) peeling using a commercial combination of Brilliant blue G (BBG, 0.25 mg/ml) with 4% polyethylene glycol (PEG).

Methods

It was a prospective, single-center study. Macular surgery was performed due to ERM (n = 18) by two experienced surgeons. Exclusion criteria were secondary ERM, previous retinal surgery and pharmacological treatment. Best-corrected visual acuity (BCVA), optical coherence tomography (OCT), and multifocal ERG (RETIscan) were assessed at baseline and three months after surgery.

Results

The BCVA improved from baseline 0.4 ± 0.13 logMAR to 0.3 ± 0.2 logMAR after three months (p > 0.05). The mean central foveal thickness was reduced from 407 ± 85 μm to 366 ± 56 μm after three months (p > 0.05). At baseline, the mean P1 amplitude (nV/deg^2^) was 53.5 ± 32.1 in ring 1 and 35.9 ± 20.1 in ring 2. Three months after surgery the mean P1 amplitude was comparable with 57.2 ± 16.3 in ring 1 and 38.0 ± 11.7 in ring 2 compared with the initial situation (p = 0.22 and p = 0.3, respectively).

Conclusion

BBG with 4% PEG can be used for ILM peeling in patients with idiopathic epiretinal membranes without any sign of short-term toxicity.

## Introduction

A macular epiretinal membrane (ERM) is characterized by fibrocellular proliferation of the internal limiting membrane (ILM) [1]. Standard surgical treatment is a pars plana vitrectomy and membrane peeling combined with removal of the ILM to remove the scaffold for myofibroblast proliferation and prevent ERM recurrence [2]. Remaining parts of the ERM at the ILM serve as a scaffold for a potential reoccurrence. Therefore, complete removal of both, the ERM and the ILM, is postulated [3]. To facilitate macular surgery, a variety of vital dyes have been developed to allow peeling of the barely visible ILM.

The surgeon's choice of dye is influenced by many factors, including color contrast, specific staining properties and most importantly potential toxicity [4]. Some dyes are thought to potentially alter the biomechanical properties of the ILM by increasing stiffness potentially facilitating peeling [5-7]. Using indocyanine green (ICG), one of the first dyes [8] for ILM peeling, is controversial because of reports about toxicity [9-11]. It was reported to exert a negative effect on the retina [12,13]. Therefore, lower concentrations and immediate washout were used to try to prevent retinal damage by ICG [14] and surgeons try to avoid using this dye if other medical devices are at hand. Vital dyes such as Brilliant blue G (BBG) [15-17] selectively staining the ILM are currently used, especially in Europe [4].

The use of heavy dyes facilitating sedimentation on the retina without the need for fluid-air-exchange has become popular in the recent past [3]. Two commercially fixed combinations are BBG (Brilliant blue G, 0.25 mg/ml) with 4% polyethylene glycol (PEG), available as ILM Blue® (D.O.R.C. Zuidland, Netherlands) and BBG (0.25 mg/ml) with Trypan blue (0.25 mg/ml) and 4% PEG, available as Membrane Blue Dual (MBB Dual) (D.O.R.C. Zuidland, Netherlands). Januschowski et al. were able to show biocompatibility for both dyes in the isolated perfused vertebrate retina organ culture model and in a retinal ganglion cell line [3]. Whereby ILM Blue seems to have a better biocompatibility which might be advantageous for repeated staining of the ILM [3]. Other possibilities to facilitate staining are adding glucose or cooling the dye. However, negative reports about e.g. aminotriarylmethanic acid violet 17 (AV17) have surfaced lately generating insecurity among retinal surgeons thus making the subject of medical devices and toxicity more important than ever [18-20]. Retinal surgeons need an on-label product at hand that minimizes risk. Recently the combination BBG (Brilliant blue G, 0.25 mg/ml) with 4% PEG was submitted for FDA approval (NDA 209569). It was the aim of this study to evaluate the clinical safety using ILM Blue in a real life clinical prospective setting.

## Materials and methods

In this prospective study 24 patients underwent surgery due to idiopathic ERM by two experienced surgeons (>4000 vitrectomies). They were evaluated from July 2018 to November 2018. Only one eye of the patient was included into the study. Six patients were excluded because of incomplete follow-up. The study followed the tenets of the Declaration of Helsinki and was approved by the local ethical committee (ethics committee of the “Ärztekammer des Saarlandes” 252/15, study registration DRKS00014597). Informed consent was obtained from all patients before enrolment in the study.

Exclusion criteria were secondary ERM (e.g., retinal detachment, diabetic retinopathy, venous occlusion, uveitis). Patients with previous retinal surgery, high myopia (>6 diopters) or previous pharmacological treatment for ERM (e.g., ocriplasmin) were excluded from the study.

Best-corrected visual acuity (BCVA), optical coherence tomography (OCT) (Heidelberg Engineering, Heidelberg, Germany), and multifocal ERG (RETIscan; Roland Consult Elektrophysiologische Diagnostik Systeme, Wiesbaden, Germany) were measured at baseline and three months after surgery. The photoreceptor status on OCT was classified into two groups: intact and disrupt. An intact photoreceptor line was interpreted as a regular continuation of the inner segment/outer segment junction (ISOS).

**Figure 1:**
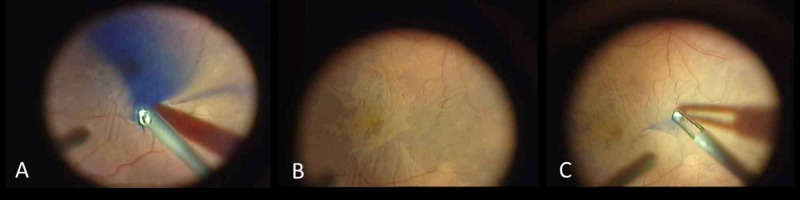
Intraoperative steps of ILM peeling with ILM Blue. A) ILM Blue, which sediments at the posterior pole. B) Staining of ILM Blue unevenly due to the presence of the epiretinal membrane. C) Maculorrhexis: ILM flap grasped with an end gripping forceps. ILM: Internal limiting membrane

We performed a standard 23-gauge suture-less vitrectomy with ILM Blue® (BBG 0.025%+ 4% PEG D.O.R. C. Zuidland, Netherlands) assisted ERM/ILM removal. A standard ophthalmic operating microscope (Lumera 7 CS microscope, Carl Zeiss Meditec Inc., Germany) and a regular vitrectomy setup (Eva, D.O.R.C., Zuidland, Netherlands) with endoillumination (80%) was used. During the procedure we used a two-dimensional cutter (TDC Cutter 23G D.O.R.C., Zuidland, Netherlands) set to 8000 cpm during core vitrectomy (maximum vacuum 450 mmHg), peripheral vitrectomy (maximum vacuum 250 mmHg) and shaving for all procedures. After the core vitrectomy we performed a detachment of the posterior hyaloid followed by a thorough peripheral vitrectomy. Afterwards ILM Blue (D.O.R.C. Zuidland, Netherlands) was injected into the BSS filled eye and removed after a staining period of 30-60 seconds (Figure 1). Specifications of 0.5 ml ILM Blue® are: Brilliant Blue G: 0.125 mg (97% purity, PEG: 4% PEG 3350, concentration: 0.25 g/l, pH-value: 7.3-7.6, osmolality: 301-369 mOsm/kg H2O (DORC, Netherlands). If necessary, phacoemulsification and intraocular lens (IOL) implantation was performed. Triamcinolone staining was not used during surgery. The ILM was peeled around the foveal region using end gripping forceps (23G forceps, ILM End-Gripping, Vitreq, Eire UK) with a radius of approximately 1.5 disc diameters. Air-fluid exchange was performed followed by an injection of 20% sulfur hexafluoride (SF6) if necessary (3/18).

Descriptive and statistical analysis were performed using SPSS software (version 12.0 for Windows; SPSS, Inc., Chicago, IL, USA) and presented in terms of mean, standard deviation (SD) and range or percentage, as appropriate. Comparison of data was performed using the t-test, and chi-square test, as appropriate. Non-parametric data were evaluated using Wilcoxon-Mann-Whitney Test, Wilcoxon Signed Rank Test, and Kruskal Wallis Test, as appropriate. Associations between non-continuous variables were analysed using Fisher’s exact Test. Statistical significance was considered with a p-value of < 0.05.

## Results

Macular surgery was performed due to ERM (n = 18). The baseline characteristics are shown in Table 1. The mean (± SD) age of the patients was 72.7 ± 6.3 years (six men/12 women). Four of 18 eyes underwent combined cataract surgery, which had no significant influence on the postoperative visual acuity after three months (p = 0.31). None of the patients required further treatment during the three-month follow-up.

**Table 1:** Baseline characteristics and postoperative outcome three months after surgery in patients with idiopathic epiretinal membrane (ERM) (n = 18). BCVA: Best-corrected visual acuity; SD: Standard deviation; logMAR: logarithm of the minimum angle of resolution.

	Preoperative (Mean ± SD)	Postoperative (Mean ± SD)	p-value
BCVA in logMAR	0.4 ± 0.13	0.3 ± 0.2	>0.05
Central foveal thickness in µm	406.6 ± 85.4	366.2 ± 56.2	>0.05
Photoreceptor status, intact:disrupt	11:7	10:8	>0.05

The improvement in visual acuity was ≥2 lines in 11 of 18 eyes (61%). No patient had a worse visual acuity postoperatively. Postoperative visual acuity showed no correlation between the two surgeons (p = 0.36). All patients had preoperative subjective metamorphopsia, which significantly improved in the final control after three months (16/18) (p < 0.05).

After surgery, the central foveal thickness was improved in all cases. The mean central foveal thickness was reduced from 407 ± 85 μm to 366 ± 56 μm after three months (p > 0.05). Preoperatively, 7/18 patients showed a disrupted photoreceptor status, which improved postoperatively in only one case. In two cases a disrupted photoreceptor status developed postoperatively, which had a significant influence on the postoperative visual acuity after three months (p = 0.048).

At baseline, the mean P1 amplitude (nV/deg2) was 53.5 ± 32.1 in ring 1 and 35.9 ± 20.1 in ring 2. Three months after surgery the mean P1 amplitude was comparable with 57.2 ± 16.3 in ring 1 and 38.0 ± 11.7 in ring 2 compared with the initial situation (p = 0.22 and p = 0.3, respectively). There was a statistically significant correlation between P1 amplitude of ring 1 and visual acuity and retinal thickness at baseline and three months after surgery (p < 0.05).

Ring 1 and 2 responses of the P1 waves were significantly decreased at baseline compared with the normal fellow eyes (p < 0.05) (Table 2). There was no significant difference in the P1 response in the peripheral area (rings 3-5) and the N1 response in the whole area compared to normal fellow eyes (p > 0.05). After surgery, the P1 and N1 amplitudes at the peripheral area (rings 3-5) did not show any significant changes.

**Table 2:** P1 waves of multifocal electroretinography (mfERG) preoperative and three months after surgery.

	Preoperative	Postoperative	Fellow Eye
P1 amplitude (nV/deg²)			
Ring 1	53.5 ± 32.1*	57.2 ± 16.3*	108.7 ± 22.4
Ring 2	35.9 ± 20.1*	38.0 ± 11.7*	58.2 ± 10.5
Ring 3	28.2 ± 3.6	26.1 ± 3.5	38.5 ± 8.2
Ring 4	19.2 ± 2.7	18.3 ± 3.3	31.2 ± 4.9
Ring 5	14.5 ± 2.0	15.2 ± 2.5	20.3 ± 4.2
P1 latency (ms)			
Ring 1	45.2 ± 2.7	47.0 ± 3.4	44.9 ± 2.4
Ring 2	42.7 ± 3.4	44.7 ± 2.6	41.3 ± 2.1
Ring 3	42.0 ± 2.9	43.1 ± 2.8	41.1 ± 1.8
Ring 4	42.7 ± 2.9	44.3 ± 1.4	42.3 ± 1.9
Ring 5	43.4 ± 2.2	44.6 ± 1.9	42.9 ± 1.7

We could not show a statistically significant correlation between the amplitudes P1 and N1 (all 5 rings) and the disrupted ISOS preoperatively and three months after surgery (p > 0.05).

## Discussion

In this study, we were able to show that ILM Blue®-assisted peeling is safe in a prospective, real life clinical setting. Our data are supported by cell culture experiments in which no toxicity of BBG was described [3,21].

BBG performs a selective ILM staining with a low affinity for ERM [4,22]. This is important because the visualization and removal of the ILM is challenging as the structure is about 1 µm thick and is formed by the end feet of Müller cells and astrocytes. A specific staining of the ERM/ILM and thus an easier removal in macular surgery is important for the postoperative outcome. Thus, for example, errors such as accidental injury to the nerve fiber layer can be avoided by staining. In earlier studies, ILM Blue® has shown a slightly better biocompatibility compared to combined vital dyes, which is an advantage for repeated staining of the ILM [3]. This may be necessary especially for firmly adhering membranes. In our study, this was also shown in three cases where re-dyeing was necessary, which in turn had no significant influence on the study results.

A possible explanation of Januschowski et al. for the improved biocompatibility of ILM Blue could be that the dye solutions sold by a manufacturer place special emphasis on the purity of the ingredients and quality control [3]. It could also be argued that the presence of 4% PEG has a potential neuroprotective effect [22]. In particular, potential toxicity is a decisive factor in the choice of the vital dye and is part of the current research, as the currently used vital dyes have shown retinal toxic reactions [5,9-11,18,19]. In this study, we cannot assume a toxic effect of ILM Blue due to the stable multifocal ERG (mfERG) values after surgery.

The mfERG as an objective assessment of retinal function can be influenced by several factors including traction membrane removal, photoreceptor status, cataract progression, intentional ILM removal and the use of vital dyes. Since all phakic patients underwent simultaneously performed phacoemulsification, the factor cataract could be ruled out.

Previous reports have shown that mfERG responses decreased three months after ERM surgery with ILM peeling without significance compared to baseline [23-25]. On the contrary, our results did not show a reduced amplitude in ring 1 and 2, but rather similar values. Furthermore, we could show that there was a statistically significant correlation between P1 amplitude (ring 1) and visual acuity and retinal thickness at baseline and three months after surgery. In addition, Lim et al. and Koutsandrea et al. showed an increased amplitude even 12 months after epiretinal membrane surgery [24,26]. However, Lim et al. were able to find deposits of retinal cells in an electron microscopic examination of the ILM after ERM peeling, so that this could probably also have some effects on mfERG changes due to damage to the inner retinal layers and Müller cell dysfunction [26]. Since the impairment of the amplitude of P1 and N1 is consistent even 12 months after surgery, this could indicate that successful ERM removal does not lead to a complete restoration of modulation of synaptic transmission in the retinal neural circuit [26].

The photoreceptor status also has a decisive influence on macular function and thus on the mfERG, and this photoreceptor status is also primarily determined by the surgical procedure. Therefore, we have limited this prospective study to two experienced surgeons. We could not show a significant difference between the pre- and postoperative photoreceptor status, which argues against a decisive influence of the ILM Blue on the photoreceptor status.

The mfERG value can be influenced by numerous factors that we cannot recognize and control, e.g. the tension on the macula during membrane peeling can also affect the function of the macula [27], so that a correlation with ERM peeling may not be easy to interpret [26]. Problems in fixation stability in patients with macular abnormalities may affect mfERG results, especially for ring 1 in the mfERG concentric ring analysis [27]. However, we used RETIscan, which showed low variability due to averaging of the mfERG reactions over the hexagons with identical eccentricity [28].

The limitations of our study are the small sample size and a relatively short follow-up time, which may have led to insufficient statistical analysis. Recently it has been shown that a sample size of more than 1000 would be required to detect subtle negative effects of a vital dye [29]. However, it is in the best interest of vitreoretinal surgeons to have real life safety data early on to improve patient safety.

## Conclusions

In summary, we can nevertheless show in this study that ILM Blue® can be used for ILM peeling without showing signs of short-term toxicity. Larger controlled studies are justified to improve our understanding of the changes that can occur after ILM surgery.
